# Scientific Review Committees as part of institutional review of human participant research: Initial implementation at institutions with Clinical and Translational Science Awards

**DOI:** 10.1017/cts.2019.439

**Published:** 2020-01-27

**Authors:** Harry P. Selker, Lisa C. Welch, Elizabeth Patchen-Fowler, Janis L. Breeze, Norma Terrin, Anshu Parajulee, Amy LeClair, Arash Naeim, Rebecca Marnocha, Julie Morelli Novak, Christine Sego Caldwell, Philip A. Cola, Jennifer A. Croker, David X. Cifu, Kirsten M. Williams, Denise C. Snyder, Darlene Kitterman

**Affiliations:** 1 Tufts Clinical and Translational Science Institute, Tufts University, Boston, MA, USA; 2 Institute for Clinical Research and Health Policy Studies, Tufts Medical Center, Boston, MA, USA; 3 UCLA Clinical and Translational Science Institute, University of California, Los Angeles (UCLA), Los Angeles, CA, USA; 4 University of Wisconsin Institute for Clinical and Translational Research, University of Wisconsin-Madison, Madison, WI, USA; 5 Tufts Health Sciences Institutional Review Board, Tufts University, Boston, MA, USA; 6 Indiana Clinical and Translational Sciences Institute, Indiana University, Indianapolis, IN, USA; 7 Clinical and Translational Science Collaborative (CTSC) of Cleveland, Case Western Reserve University, Cleveland, OH, USA; 8 Center for Clinical and Translational Science, University of Alabama at Birmingham, Birmingham, AL, USA; 9 VCU C. Kenneth and Dianne Wright Center for Clinical and Translational Research, Virginia Commonwealth University, Richmond, VA, USA; 10 Clinical and Translational Science Institute at Children’s National, George Washington University, Washington, DC, USA; 11 Duke Clinical and Translational Science Institute, Duke University School of Medicine, Durham, NC, USA; 12 Oregon Clinical and Translational Research Institute, Oregon Health and Science University, Portland, OR, USA

**Keywords:** Ethics review, Scientific Review Committee, scientific quality, operational feasibility, quantitative and qualitative methods

## Abstract

**Introduction:**

Scientific quality and feasibility are part of ethics review by Institutional Review Boards (IRBs). Scientific Review Committees (SRCs) were proposed to facilitate this assessment by the Clinical and Translational Science Award (CTSA) SRC Consensus Group. This study assessed SRC feasibility and impact at CTSA-affiliated academic health centers (AHCs).

**Methods:**

SRC implementation at 10 AHCs was assessed pre/post-intervention using quantitative and qualitative methods. Pre-intervention, four AHCs had no SRC, and six had at least one SRC needing modifications to better align with Consensus Group recommendations.

**Results:**

Facilitators of successful SRC implementation included broad-based communication, an external motivator, senior-level support, and committed SRC reviewers. Barriers included limited resources and staffing, variable local mandates, limited SRC authority, lack of anticipated benefit, and operational challenges. Research protocol quality did not differ significantly between study periods, but respondents suggested positive effects. During intervention, median total review duration did not lengthen for the 40% of protocols approved within 3 weeks. For the 60% under review after 3 weeks, review was lengthened primarily due to longer IRB review for SRC-reviewed protocols. Site interviews recommended designing locally effective SRC processes, building buy-in by communication or by mandate, allowing time for planning and sharing best practices, and connecting SRC and IRB procedures.

**Conclusions:**

The CTSA SRC Consensus Group recommendations appear feasible. Although not conclusive in this relatively short initial implementation, sites perceived positive impact by SRCs on study quality. Optimal benefit will require local or federal mandate for implementation, adapting processes to local contexts, and employing SRC stipulations.

## Introduction

Adherence to ethical principles for human participant research is integral to the biomedical research enterprise [1–4]. There is international agreement that independent committees should review protocols to ensure studies respect participant rights and minimize risks, done by Institutional Review Boards (IRBs). Two of the key ethical principles for the review of human participant research are to ensure that research risks are minimized and reasonable in relation to anticipated benefits. Research review should take into consideration a study’s scientific quality and operational feasibility to ensure that a protocol can be completed and will achieve its objectives so that the fewest number of participants are exposed to risk to answer the study question, and that the study question is worthwhile to answer [5,6]. To do this some organizations have instituted Scientific Review Committees (SRCs) that typically precede IRB review, which allow focused assessment of the multiple dimensions of scientific quality and operational feasibility.

Responding to these principles and believing that SRCs could augment the quality of ethics review of human participant research, recommendations were made by the NIH Clinical and Translational Science Award (CTSA) Consortium Consensus SRC Working Group Report on the SRC Processes [7–8]. They proposed a framework across seven domains guiding how protocols are chosen for SRC review, how protocols are assessed, and the processes in place at institutions to move protocols through scientific review (see Supplemental Table S1). These guidelines stimulated some organizations to adopt SRC processes, but to date their implementation has not been studied.

The goal of this study was to assess the feasibility and impact of the recommended CTSA SRC Consensus Group framework across a variety of academic health center (AHC) research organizations with CTSAs. The study hypothesized that implementing the framework would improve the scientific quality of biomedical research protocols without a meaningful change in overall duration of ethics review (IRB and SRC, if applicable).

## Methods

### Research Design

Institution of SRC processes or modification of existing practices at the sites was led by a Coordinating Center at Tufts Clinical and Translational Science Institute (CTSI). This included guidance on interpretation and implementation of the Consensus Group recommendations and on-site meetings with implementing teams.

The study evaluated the intervention of SRCs at participating sites, using a pre–post-intervention mixed method design. Quantitative and qualitative data were collected for two 6-month study periods starting in February 2016. These were the baseline and intervention periods, with an intervening 2-month implementation period to allow sites to align SRC processes with the recommended framework. For protocols pending approval at the end of the pre- or post-study period, follow-up extended 7 weeks beyond each period to allow data capture.

Quantitative measures of the implementation of SRC processes assessed the extent to which SRCs aligned with recommended criteria and pre–post SRC member turnover as one indicator of feasibility of implementing those criteria. Qualitative data described barriers, facilitators, and recommendations.

Research protocols from each period were assessed for scientific quality. A quality review group composed of independent reviewers rated protocols on the seven recommended SRC review categories: study objectives, scientific merit/background and rationale, study design, eligibility criteria, outcomes and endpoints, analysis and sample size, and data management.

Efficiency of review was assessed by two indicators: (1) Median duration of review to achieve IRB approval was defined as days from initial submission to IRB approval, including any SRC process time but excluding time a protocol was with the investigator, and (2) overlapping effort between the IRB and SRC was operationalized as the proportion of protocols with IRB stipulations related to SRC foci, that is, scientific quality and operational feasibility.

### Participants

Academic medical research organizations with CTSAs in 2015 were invited to participate, with the goal of recruiting sites of diverse size and research portfolios. Seventeen organizations expressed interest and completed a screening questionnaire. To achieve a range of scientific review configurations at baseline, 13 sites were selected, 5 withdrew prior to the intervention, and 2 additional were added. The 10 participating sites had diverse extant situations: no SRC process (*n* = 4), scientific review processes varying across individual departments or units (*n* = 2), or centralized scientific review processes (*n* = 4). Within each selected site, all extant SRCs other than those related to cancer centers (as NIH cancer centers have requirements for an SRC review process) were eligible to participate.

### Procedures

During the baseline period, sites continued with their ethics review procedures in place prior to this study. The research team prioritized 15 recommended criteria in 5 domains for sites to seek to implement (Table 1). Each site selected the criteria that were feasible to implement locally during the 2-month implementation period and apply during the intervention period. Activities differed across sites but often included creating or modifying policies and workflows, training SRC members, and communicating with organizational leadership and/or investigators. Two sites modified existing informatics systems to support new workflows or deliver study data.

**Table 1.** Change in proportion of Scientific Review Committees (SRCs) that met or exceeded prioritized criteria for policies and practices

Criterion	SRCs				
	Baseline *n* = 30*		Intervention *n* = 29		Net change
	*n*	%	n	%	%
Review content					
Review for scientific quality	26	86.7	29	100.0	13.3
Review for local operational feasibility	25	83.3	24	82.8	−0.5
Locally reviewed protocols	26	86.7	29	100.0	13.3
Centrally reviewed protocols	25	83.3	24	82.8	−0.5
Protocol eligibility					
All protocols are eligible for SRC review or exemption criteria are limited to recommended types of protocols	23	76.7	26	89.7	13.0
Protocols that fit criteria for exemption can still be reviewed (includes SRCs that do not exempt protocols)	25	83.3	28	96.6	13.3
Institutional Review Board (IRB) and/or institutional officials may forward to the SRC any protocol at any time	21	70.0	26	89.7	19.7
Reviewers per protocol					
At least one medical review	26	86.7	29	100.0	3.0
Statistician not also acting as a medical reviewer	4	13.3	15	53.6**	40.3
Content experts as needed	24	80.0	28	96.6	16.6
Reviewer qualifications					
Have requisite expertise	25	83.3	29	100.0	16.7
Not on research team	22	73.3	27	93.1	19.8
No conflict of interest	21	70.0	25	86.2	16.2
Is available to perform review in timely manner	25	83.3	28	96.6	13.3
Is willing to undertake the task	25	83.3	28	96.6	13.3
Related institutional policies					
Coordination of the SRC with the IRB	25	83.3	28	96.6	13.3
IRB has access to SRC review	24	80.0	27	93.1	13.1

* Four sites without an SRC at baseline are included in the denominator as one SRC per site; each of these sites established a single SRC process at intervention.

** Data are missing for one SRC. The denominator for this proportion is 28 instead of 29.

#### Quality review group

An independent review group from non-participating CTSAs was assembled to assess research protocol quality, including 28 as content reviewers and 20 as statistical reviewers. Each of the 120 protocols was separately scored by 1 content reviewer and 1 statistical reviewer for the 7 recommended categories and was given an overall score on a 9-point scale. If the two reviewers’ overall scores differed by three or more points, they were asked to discuss and revise their scores, if on reconsideration it was felt appropriate. Reviewers were blinded to protocol study period, investigator names, and originating site.

### Data

#### Quantitative data

Data were collected on each participating SRC; sites with multiple SRCs were instructed to treat each as distinct if they followed different policies and procedures. Measures of alignment with each of the 15 prioritized criteria were asked at the beginning of the baseline and intervention periods. If an SRC made modifications during the intervention period, data on alignment were re-assessed at the end of intervention. Each SRC also reported the number of members at the start of each study period and the number added or lost during each period. Protocol-level measures included scientific quality scores, time in the SRC and IRB, and type of stipulations (scientific quality, feasibility, other). Quantitative data were collected and managed using the Research Electronic Data Capture (REDCap) tools hosted at Tufts Medical Center (versions 5.10–6.10.1) [9]. Data managers at each site received training and technical support by the Coordinating Center.

#### Qualitative data

Two semi-structured interview guides were developed, one for local SRC champions and implementers, and the other for SRC and IRB chairs. Topics included barriers and facilitators for implementing the framework, challenges or concerns, organizational support, communication between the SRC and others (e.g., IRB, investigators, deans), and recommendations for other organizations seeking to implement the framework.

Two investigators conducted interviews by telephone. With participant permission, interviews were recorded and transcribed verbatim. To assure transcription accuracy, the study team reviewed a subset of transcripts.

### Sampling

#### SRC level

All participating SRCs were included in analyses to assess alignment with the prioritized criteria. Analyses to assess SRC member turnover included SRCs that implemented a modification at intervention (including new SRCs) and their corresponding SRCs at baseline (if applicable), provided that they submitted data of acceptable quality.

#### Protocol level

Clinical/biomedical protocols with human participants were eligible if review by an IRB began during the study periods. Excluded were protocols exempt from IRB review or reviewed by an expedited procedure. Protocols reviewed by an external relied-upon IRB were excluded because it was not feasible for local IRBs to collect detailed data about them.

Protocol-level analyses included only those from sites that implemented a modification to prioritized criteria, had sufficient data to analyze (at least one intervention-period protocol), and provided data of acceptable quality. Protocols that did not receive IRB approval within the study follow-up period were excluded from all analyses except the analysis of duration of ethics review, which excluded protocols withdrawn from the review process.

For quality review analyses, baseline protocols were included only if the site responded affirmatively to the question: “If this protocol came in today, would it get SRC review/the specific modification to SRC review?” Due to lower than expected volume of eligible protocols at 3 sites eligible for inclusion in quality review analyses, all intervention protocols were included at eligible sites with fewer than 12, and baseline protocols were randomly sampled to match the number at intervention. At eligible sites with more than 12 eligible protocols during intervention, equal numbers of eligible protocols were randomly selected from the two time periods to obtain the target sample size (60 in each period).

#### Qualitative interviews

Purposive sampling was used to achieve a range of perspectives at each site, including a local “champion” of implementing the recommended SRC framework, an implementer, the SRC chair, and the IRB chair. At sites where no SRC made a modification, only the SRC champion was interviewed.

### Analytic Strategy

#### Statistical analysis

To assess alignment with the prioritized SRC criteria (Table 1), each SRC received a score indicating the extent of alignment within each of the five domains. Six points were possible within each domain, yielding a range of 0–30 points (see Supplemental Table S1). For each SRC, the overall alignment score was tabulated for baseline and for intervention. For each prioritized criterion, the proportion of SRCs aligned was calculated for both time periods and the net change determined.

To assess SRC member turnover, the number of members added and lost at each SRC per study period was calculated as percent change from the initial number of members for that period. Summary statistics of percent change (mean, median, range) were calculated for each study period and compared.

For each protocol in the quality review sample, the overall assessment scores of the two reviewers were averaged for a single score. Differences in scores between baseline and intervention were tested using the two-sample *t*-test. Chi-square tests were used to compare the proportion of protocols with resubmissions and with stipulations. Time for completion of ethics review (in calendar days) was assessed using a Cox model with adjustment for site as a fixed effect. Protocols were censored if they had not received ethics approval by the end of the study period. A time-dependent covariate was included to account for non-proportional hazards.

#### Qualitative thematic analysis

De-identified transcripts of semi-structured interviews were uploaded into Dedoose™ analytic software for coding and analysis. An initial codebook was developed deductively from the interview guide and then inductively revised in an iterative process to more closely reflect emergent themes. This process entailed consensus coding of 10 transcripts in which 2 analysts individually coded 3 rounds of transcripts and met to compare coding. At that point, the analysts were applying codes with >85% consistency. The remaining transcripts were divided and coded independently, and the initial 10 transcripts were reviewed using the finalized codebook [10]. Coders met to discuss concerns and reach consensus in cases of uncertainty. Data were analyzed using a thematic approach by examining coding frequency and thoroughly examining all quotations within the most frequent codes [11]. For major themes, site-level analyses were conducted to provide context, including the number of sites for which each theme applied.

## Results

### Feasibility of CTSA SRC Consensus Group Recommendations Implementation

Ten sites participated during both study periods, spanning three types of extant SRC processes: four sites did not have SRC processes, four had a single SRC process conducted by a centralized group or by individual departments, and two sites had multiple extant SRC processes that participated in the study (*n* = 7 and *n* = 15). Within the three types, SRC alignment at baseline, when applicable, and modifications at intervention varied without a clear pattern according to site type (Supplemental Fig. 1).

**Fig. 1.**
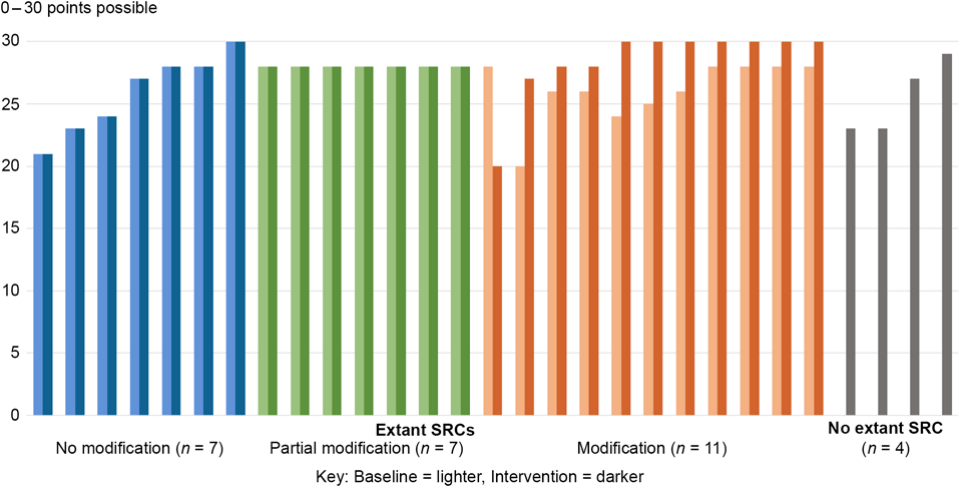
Alignment of Scientific Review Committee (SRC) processes with prioritized criteria at baseline and intervention by category of modification.

*Note*: Intervention data are missing for one criterion at one SRC (2 points). Sites with no extant SRC at baseline do not have data for that period. At one site, two baseline SRCs merged into a single intervention SRC; the baseline score represented is the average of the two baseline SRC individual scores.

Across sites, the 26 extant SRC processes at baseline were relatively well aligned with the prioritized criteria (Fig. 1). Alignment scores ranged from 20 to 30 out of a possible 30 points. Incorporating the 4 sites with no SRC process (with alignment scores = 0), the average baseline score was 23.0 points.

At intervention, each of the four sites without an extant SRC implemented an SRC, five sites with extant SRCs further aligned with prioritized recommendations, and one site with an extant SRC did not make modifications. Across sites, 29 different SRC processes participated. While the four sites without extant SRC processes each initiated a single SRC process, two distinct SRC processes at another site were combined into a single process at intervention.

Among the 29 participating SRCs during the intervention period, 14 (48%) were new or made modifications in prioritized criteria that were substantial enough to increase alignment scores. Seven (24%) made modifications toward more alignment but not sufficient to increase the score, seven (24%) did not modify the prioritized criteria during the study period, and one (3%) became less aligned with the prioritized criteria. Most SRCs that implemented modifications did so at the beginning of the intervention period as planned, but three implemented them between 1 and 4 months into the intervention period.

Overall, alignment scores at intervention averaged 27.3 out of a possible 30 points, an increase of 4.3 points from baseline. Most prioritized criteria saw a modest overall increase in the percent of SRC processes aligned (Table 1). All SRCs aligned with reviewing protocols for scientific quality and, for protocols reviewed locally, operational feasibility. For protocols reviewed by an external IRB, local operational feasibility was assessed less often, with very little change from baseline to intervention (83.3% and 82.8%, respectively). One criterion – assuring that the statistical reviewer was not also a medical reviewer – simultaneously saw the largest increase in the percent of SRC processes aligned (a change of 40.3%) and remained the criterion with which SRCs were least often aligned at both baseline and intervention (13.3% and 53.6%, respectively).

Five extant SRCs and eight intervention-period SRCs were eligible for analysis of member turnover. During the baseline period, SRCs experienced an average 0.4% decrease in members (median: 0, range: −2.2% to 0). During intervention, SRCs saw an average 6.0% increase in members (median 1.0%; range: 0–22.2%), and no members were lost.

### Barriers and Facilitators to Robust Implementation

The site-level qualitative analysis was based on 36 semi-structured interviews: 4 respondents from each of 8 sites, 3 respondents from 1 site, and 1 respondent from the site where planned modifications to the single SRC were not implemented during the study period. Respondents spanned the four targeted roles (a local implementation champion, an implementer, the SRC chair, and the IRB chair).

#### Barriers to implementation

SRCs experienced three main barriers to implementation of the CTSA SRC Consensus Group recommendations (Table 2). First, respondents at half of the sites expressed uncertainly about long-term sustainability of implementing the recommended SRC process due to resource requirements and vulnerabilities of local mandates. Long-term resource challenges included (i) securing sufficient staff to avoid delays in review time and/or expand implementation across the organization, particularly for decentralized SRC processes conducted by departments, and (ii) sustaining consistency when funding depended on CTSA grant cycles.

**Table 2.** Barriers to robust implementation

Difficulties sustaining the Scientific Review Committee (SRC)
Securing resources and staff
*Concern voiced by four of eight sites addressing the topic*
At some point, this is going to cost real resources from somebody. – *Local SRC champion*
Vulnerabilities of a local mandate
*Issue voiced by 4 of 10 sites addressing the topic*
We have to [run a tight ship] because I know that if we screw up just once, or someone has to wait, the feedback, it could end this, until we’re fully entrenched. – *Local SRC champion*
I think the higher ups want to see something happen, but they need support before they can mandate anything… We’re working towards that, but we need some data to prove that it’s necessary before we can actually mandate it. *− Implementation point person*
**Limited reach of SRC authority** *Lack of authority reported by four of nine sites addressing the topic*
[A]t the end of the day, [the SRC] can’t tell someone they can’t do their research project. Only the IRB [Institutional Review Board]…, higher up, can do that. …But, I do know that the IRB reviewers take the scientific reviews seriously and utilize those in their reviews. – *Implementation point person*
[Without local authority to require investigators to respond to SRC review,] “I mean, we really need the mandate to make it [the SRC process] function appropriately” *− Implementation point person*
**Difficulties demonstrating anticipated impact** *Positive impact anticipated at eight of nine sites, but concerns with demonstrating it*
Lack of authority
The SRC committee [for] a certain subset of the studies that they’ve reviewed have found legitimate – that they would put into a moderate to major [revisions] category. So if the metric is “Can an SRC committee find areas for improvement?” I think the answer to that is probably “Yes.” So, whether or not an SRC process has actually improved a protocol or not…is hindered a bit because this is a non-mandated process. – *Local SRC champion*
Short evaluation period
A lot of our studies haven’t actually gone through to the IRB yet; they’re still working on their protocol. So it’s hard to really see the impact yet. – *Local SRC champion*
What I would like to do is see how many trials, for instance, the proportion of trials that were approved by the IRB before the SRC started and were not feasible, and were ultimately closed for lack of accrual or lack of whatever. …But that’s going to be years down the road. – *SRC Chair*

Although not asked directly to comment on whether SRC processes should be mandated, respondents at four sites raised the topic. At three of these, SRC review was required by the organization, creating a local mandate for implementation. At two of these sites, respondents noted that a firm local requirement facilitated implementation, but the third site characterized its new local SRC requirement as potentially vulnerable. Despite substantial outreach and education that created widespread buy-in across departments and schools, the SRC champion believed they needed to “run a tight ship” to maintain organizational support. The fourth site reported an even more significant vulnerability of relying on a local requirement: convincing local leaders that review beyond that of the IRB is necessary. In the absence of an external mandate, local leaders at this site required evidence of the value of SRC review before agreeing to robust implementation.

Second, the authority granted to SRCs varied across sites. Among the nine sites for which respondents addressed this issue, SRCs at five were vested with authority to require investigators to address comments before the protocol could move forward. At two others, although the SRC did not have authority to withhold protocol approval, the IRB could, and did, require investigators to address SRC comments. In this approach, the potential impact of the SRC depended on its relationship with the IRB, which presented a challenge when a small number of investigators questioned the SRC’s legitimacy.

The remaining two sites limited SRC authority even further, in different ways. At one, investigators were not provided comments from the newly implemented statistical review and therefore did not have an opportunity to revise protocols based on these comments. At the other site, investigators did not have to respond to SRC comments in order to attain IRB approval; the IRB could request SRC review, which occurred for 8% of protocols, but did not otherwise consider SRC comments.

Third, respondents at eight of the nine sites that addressed the SRC’s impact reported that the SRC had, or was expected in time to have, a positive effect. However, they also anticipated that demonstrating SRC impact through the study would be difficult for three reasons. For SRCs lacking authority to require investigators to address their comments, their recommendations were not incorporated into protocols. Particularly for sites that implemented modifications later than intended, the short timeframe and low volume of protocols during the intervention period presented a challenge for assessing impact. In addition, this study was not designed to capture more consequential long-term impacts, such as whether studies met accrual goals, met reporting requirements for clincialtrials.gov, or produced high-quality publications.

Further challenges were related to operationalizing a new or modified process (Supplemental Table S2). Four operational challenges were reported by at least two sites each. New or amended workflows required immediate additions to staff and/or their responsibilities. Supporting biostatistical review was a concern for one-third of the sites. In addition, a new or modified process also required training or retraining reviewers, administrative staff, and investigators. Additionally, with two groups reviewing protocols, expectations needed to be aligned. For investigators, this meant preparing them for two rounds of revisions. For reviewers, aligning expectations about the scope and purpose of the IRB and SRC reviews was important for the two groups to function cohesively. Lastly, most sites that attempted to create or modify an informatics system to facilitate SRC processes reported challenges in implementation.

Two anticipated concerns did not materialize as expected (Supplemental Table S3). Respondents at every site discussed concerns about “pushback” from investigators, but such resistance did not manifest to the extent anticipated. Respondents’ perspectives on the reasons for this varied. At three sites, respondents explained that the new or modified process had not yet impacted all investigators. More commonly, respondents reported strategies to minimize investigator pushback, including proactive communication, reassuring investigators that SRC review would facilitate IRB approval, and instituting workflows to ensure efficient SRC review.

Concerns about delays to review time also were widespread. Respondents varied in their perception of actual delays, and study results on this topic are reported below.

#### Facilitators for implementation

Respondents identified four main facilitators for implementing the recommended SRC framework (Table 3). Broad-based, clear, and open communication was key. Respondents at nine sites noted that communicating with investigators and research teams was important both prior to and during implementation. Prior to implementing new or modified SRC processes, education and outreach to the local research community fostered cooperation and support. Sites crafted messages to address investigator interests and concerns – that is, the value of review for scientific quality and local feasibility, and the importance of efficiency and avoiding delays. During implementation, communication with individual investigators about specific protocols helped to facilitate needed responses. Additionally, respondents at eight sites reported that communication with the IRB facilitated SRC processes and streamlined ethics review. Open communication was fostered by creating or leveraging an existing administrative connection or shared leadership position.

**Table 3.** Facilitators

Broad-based communication *Voiced by 10 of 10 sites*
Having a clear and concise message about the importance of a SRC [Scientific Review Committee] and the mission of it and the potential impact to the overall trajectory of the approval of their project and protocol, I think that’s really important. – *Implementation point person*
There has to be good communication to the PI [Principal Investigator]. There needs to be clear communication about what changes need to be made [to a protocol]… I think that people need to be available to the PIs to discuss things. *− Institutional Review Board (IRB) Chair*
[The relationship between the IRB and SRC] is very good. …We worked on this whole process together. We’ve sort of trouble-shooted wherever the issues might be. *− SRC Chair*
**Presence of external motivator** *Voiced by 7 of 10 sites*
[Without being on a timeline for the study,] I don’t know that it would have ever gotten done. …It’s easier to get buy-in when you have like a research study. People understand those deadlines. *− Implementation point person*
[T]he reason we implemented the additional [statistical] review was because it was part of the [study] protocol. I’m not sure we could have done it otherwise. I’m not sure our staff would have agreed to it. – *Local SRC champion*
**Senior-level support** *Voiced by 6 of 10 sites*
[O]ur PI of the CTSA grant is extraordinarily committed and really always accessible and wants to very much help. The institution of the whole…didn’t perceive this perhaps as the highest priority or it probably would have been implemented a long time ago. …[I]t was a culture change for them. *− SRC Chair*
And we had a lot of support from the institution. We got them ready in time for us to start the intervention. That has all gone pretty smoothly. – *Local SRC champion*
It [implementing the SRC] could not have been done without the correct leadership. I mean if I didn’t have [Colleague], who is the number one researcher at [Institution]…backing this, nothing would have happened. *− Implementation point person*
**Committed reviewers** *Voiced by 6 of 10 sites*
[O]ur approach to the SRC [reviewers] was directly having the Chair approach people that we targeted because we thought they would be thoughtful about it. It wasn’t something that they were forced to do against their will but invited to do and they’re doing it because they’re genuinely committed and interested in the process. – *Local SRC champion*
A lot of us have been here a long time, so we knew a lot of the faculty and those that would be kind of good reviewers… [I]nternal…networking allowed us to identify people…with the appropriate background. …And then we did ask them specifically, “Do you have time to commit to this?” *− Implementation point person*

Second, respondents at seven sites indicated that the study, as an external motivator, provided a rationale for SRC review, a recommended structure, and a firm deadline. Third, having the support of a respected champion or leader at the institution was identified by six sites as providing needed legitimacy for implementation. Fourth, in order for the SRC process to add value, the content of the review needed to be of high quality. Respondents at six sites noted that the willingness and buy-in of SRC reviewers required careful recruitment. As noted above, recruiting statistical reviewers with the time and willingness to commit was particularly challenging for some sites.

### Effectiveness of Recommended SRC Framework

#### Scientific quality of protocols

Five SRCs, one at each of five sites, met eligibility criteria for inclusion in quality reviews. Three were new SRCs with no extant process, and two had a single SRC process. Two factors related to implementation led to inconclusive results about whether scientific quality changed across study periods. More than 70% of protocols for this analysis were from two higher-volume sites, and both of these sites had extant SRC processes that were largely aligned with most of the prioritized criteria. This reduced the likelihood of a detectable difference in protocol quality. Moreover, SRC authority at the two higher-volume sites was low, and investigators were not required to respond to SRC stipulations. A sensitivity analysis that excluded protocols from these 2 sites yielded a sample size of 17 protocols at each study period – too small to draw conclusions. Consequently, although results showed no difference between baseline and intervention (Supplemental Table S4), the question of whether a difference would emerge were investigators required to address SRC stipulations could not be answered with confidence.

#### Efficiency of ethics review

**Fig. 2.**
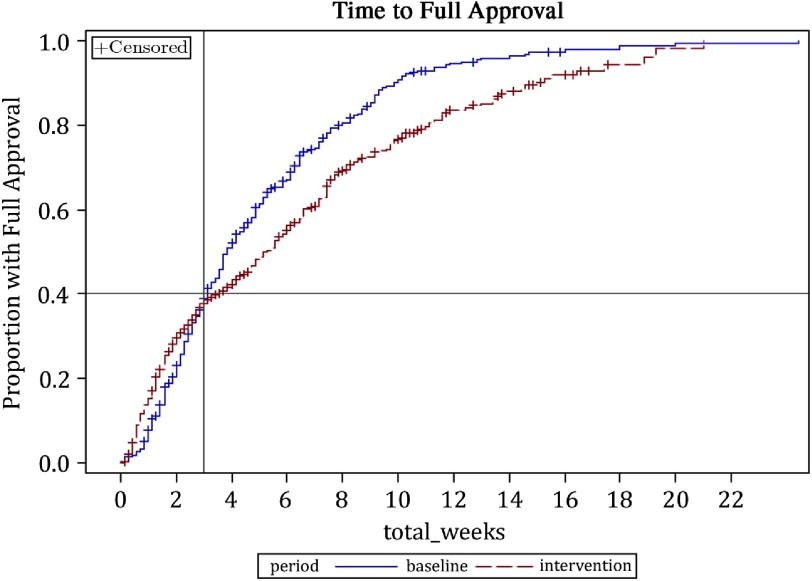
Difference in overall rate of approval by time period.

Eligible protocols originated from seven SRCs across seven sites: four with no extant SRC process and three with a single SRC process at baseline. The overall duration of ethics review (IRB and SRC, if applicable) increased for a subset of protocols (Table 4). In both time periods, about 40% of protocols were approved within 22 days. For the 60% of protocols still under review after 22 days, the rate of approval was slower in the intervention period (Fig. 2). Additionally, there was an overall increase in median duration of IRB review (24 vs. 30 days, respectively), with some protocols affected more than others. While IRB review duration during both study periods was shorter for protocols that had SRC review than for those without it, IRB review duration among protocols with SRC review was longer in the intervention period than baseline.

**Table 4.** Duration of ethics review among all eligible protocols submitted by time period (net time protocol is with investigator)

Review type	Days (median, 95% CI)		Hazard ratio**	95% CI	*p*-value
	Baseline	Intervention			
Overall (IRB and SRC, if applicable)	*n* = *415* *	*n* = *411* *			
	27	37	<22 days: 1.12	0.90, 1.41	0.3
	(25, 30)	(32, 42)	≥22 days: 0.62	0.50, 0.78	<0.0001
IRB only	*n* = *414* *	*n* = *399* *			
All protocols	24	30			
	(21, 27)	(23, 25)			
Protocols with SRC review	5	21			
	(4, 6)	(14, 32)			
Protocols without SRC review	35	36			
	(31, 41)	(29, 42)			
SRC only	*n* = *126* *	*n* = *226* *			
	8	9			
	(6, 9)	(8, 11)			

CI = confidence interval.

* Protocols censored because not yet approved: overall review: baseline = 64, intervention = 109; Institutional Review Board (IRB) review: baseline = 63, intervention = 72; Scientific Review Committee (SRC) review: baseline = 1, intervention = 44.

** Result adjusted for site as fixed effect.

Compared to baseline, the proportion of protocols requiring resubmission to IRBs was higher during the intervention period (83.5% and 92.7%, respectively, *p* = 0.0002), and this was driven by protocols from sites with no extant SRC process (79.4% and 89.5%, respectively, *p* < 0.0001). The increase in IRB resubmissions occurred in conjunction with a statistically significant decrease in the proportion of protocols with IRB stipulations related to local feasibility (13.7% vs. 5.8%, *p* = 0.015). The proportion of protocols with IRB stipulations related to scientific quality was not statistically different across study periods.

### Site Recommendations

Respondents made four recommendations for implementing the recommended SRC process (Table 5). At all sites, respondents discussed designing processes that are effective for local contexts. Although the CTSA Consensus SRC recommendations were useful guides, especially for organizations without prior experience implementing scientific review, operationalizing those recommendations amidst local processes required flexibility.

**Table 5.** Site recommendations and illustrative quotations

Design processes that are effective for local contexts
[E]ach university is a little different, and [the working group document] is a general plan of how this might be able to be integrated in. People have to find their own ways in which they can utilize it effectively to ultimately improve research and human social protection*. − Institutional Review Board (IRB) Chair*
[I]f I were designing this process to be as efficient as possible, I would design a bio-statistical review for these studies prior to IRB review. I would limit it to that. …That’s the piece that was most missing from [our] current process. *− SRC Chair*
[To recruit reviewers], we used persuasion and the support of the department chair to say this is part of your community responsibility. …That approach might or might not work at other institutions…[depending on] their culture… – *Local SRC champion*
**Invest in building buy-in through communication, or consider establishing a mandate**
I would make sure that all the stakeholders not only want this but…show up to meetings and put their name on it. … If the people that are important at your university…aren’t talking about it, I do think that could be a problem because this does require a lot of institutional buy-in on every level. *− Implementation point person*
[D]o a lot of ground swell education of their colleagues. Get a grand round slot. Do some ad hoc talks and meetings. – *SRC Chair*
I would tell them that it was critically important to get the Scientific Review Committee people together sooner than later…so that the people at [that] level felt that they had some influence or input into those process changes. – *Local SRC champion*
[T]ry to figure out a way of adding value to the process…[and] get to a place where they can mandate it. – *Local SRC champion*
**Allow time for thoughtful planning and sharing best practices**
[I would recommend] to take some time… I think for us it was key that we were organized, we had a plan in place. *− Implementation point person*
[In retrospect], I probably would have pushed for the IRB to do a lot more of this electronic part ahead of time. *− Implementation point person*
[Have] all of the people who were involved across the departments together to hear about [the recommended SRC process] and share best practices… – *Implementation point person*
**Ensure strong, clear procedural ties between the SRC and IRB**
I would suggest that having an open good relationship with the IRB office is really important. And that at the outset you really need to identify where the handoffs are and also who has the ultimate authority here. *− Implementation point person*
And make sure that your reviewers understand really what the mission of your committee is, and that they’re not reviewing a grant [application] and that they’re not reviewing an IRB submission. – *Local SRC champion*

Incorporating local context was particularly important for recruiting committed reviewers and maximizing efficiency. The content expertise needed for scientific review varied related to an organization’s research portfolio and its recruiting and retaining of reviewers (particularly statisticians); different strategies and resources were required depending on the local situation and culture. To maximize efficiency, respondents recommended three approaches that would vary according to local context: integrate the SRC process within local IRB procedures, establish locally feasible procedures to avoid delaying IRB approval, and focus on aspects of review that would provide the most value and not duplicate other reviews at an organization.

Second, respondents recommended investing in building and sustaining buy-in through communication, and/or considering establishing an external or local SRC mandate. All sites participating in the study volunteered to implement the recommended SRC processes. In this context, they took varying approaches to obtaining cooperation from investigators. Based on their experiences, respondents at 9 of the 10 sites stressed the importance of engaging and communicating with stakeholders. Support and/or cooperation were needed from institutional leaders, investigators, and IRB and SRC staff members. Two key themes to address were (i) the value of SRC review to the organization and the individual research study, and (ii) strategies that would be taken to ensure as efficient a process as possible.

Respondents at two sites recommended some type of mandate, internal or external, for fully implementing an SRC. At one of these sites, the leadership communicated its support of the study, which facilitated implementation. At the second site, organizational leaders were not in the position to require SRC review without support from investigators, making an external mandate necessary for full implementation.

Third, respondents at seven sites discussed the importance of taking time to thoroughly prepare for implementation and learn about best and promising practices from others. This included ensuring that written policies and, if possible, automated workflows were in place prior to beginning reviews of protocols. Particularly for sites with multiple SRCs, respondents recommended establishing consistent policies in advance and sharing best practices.

Finally, respondents at 6 of the 10 sites emphasized the importance of ensuring strong, clear procedural ties between the SRC and IRB. This included integrating procedures, establishing open communication, and assuring clarity of responsibilities and authority of each body.

## Discussion

This initial study of implementation of the CTSA Consortium framework for SRC processes at 10 organizations with CTSAs yielded insights that will assist in establishing new, or enhancing extant, SRC processes at clinical research centers. With one exception, sites had success in aligning at least one SRC process more closely with the framework during the study period, and the quantitative and qualitative assessments done in this study suggest approaches for creating new SRC processes, or modifying extant processes, to better align with the recommendations. In this context, there are lessons to be learned from the barriers that sites encountered that slowed or prevented robust implementation and from site recommendations to inform broader dissemination.

Absence of a clear local mandate for an SRC was a substantial barrier. While senior-level support and a firm local requirement for SRC review facilitated implementation for some sites, relying on local mandates to spearhead SRC review meant that the resources and/or will to sustain it could fade over time or, as was the case for one participating site, never fully materialize. In the context of relying on local mandates, many sites invested substantial time and effort in broad-based communication with organizational leaders and investigators.

Related to the strength of local mandates, SRC authority varied. SRCs at only about half of the sites had authority to require investigators to address their stipulations. Others either relied on the IRB to decide which SRC stipulations to enforce or, as with two sites, investigators were not required to address them.

Another key barrier was securing sufficient staffing, and this was particularly difficult with statistical reviewers. Sites anticipated this challenge to extend beyond the short-term study, raising an important resources issue that impacts long-term sustainability of SRC processes.

Despite barriers to implementation, evidence suggested a positive impact on protocol quality. Although statistical results assessing change in protocol quality were inconclusive, respondents with firsthand knowledge of SRCs’ stipulations during the intervention period reported that they had, or were expected to have with more time, a positive impact on protocols.

Additionally, available evidence suggests that the IRB gave more scrutiny to a subset of protocols in the intervention period. Anticipated delays in review time did not materialize to the extent expected, as overall review duration did not change for the two-fifths of protocols approved within 3 weeks. Yet, those still under review after 3 weeks and those referred for SRC review had longer IRB review times. At the same time, a higher proportion of protocols were resubmitted to the IRB, particularly at sites without an extant SRC, and the content of IRB stipulations during implementation focused less on operational feasibility.

Taken together, these findings of more IRB scrutiny for some protocols may indicate that some IRBs deliberated on, and, in some cases where SRCs lacked direct authority, enforced SRC comments related to scientific quality, necessitating slightly longer review duration. Although the recommended framework gives direct authority to SRCs, which lessens the burden on IRBs, in the absence of institutional support for SRC authority, IRBs would need to adopt an enforcement role. In practice, this alternative approach required a close working relationship between the SRC and IRB, and at some sites it did not result in investigators addressing SRC comments. Requirements to ensure effectiveness of this approach need further consideration.

Recommendations from participating sites highlighted the importance of local context. To implement the recommended criteria to provide the best opportunity to improve research protocols, investments of time and resources were required. This was crucial for engaging organizational leaders and IRB and SRC members, and for minimizing resistance from research teams. Absent the ability to invest in this process, an external motivator or mandate may be needed. Allowing local site flexibility and more time for organizations to respond to the recommendations of the CTSA SRC recommendations and design processes that are effective locally would support successful, efficient implementation. Although ties between IRBs and SRCs varied by local context, clear procedural ties and responsibilities across the two entities – including but not limited to which entity was authorized to enforce SRC stipulations – were important.

To put such efforts and investments in context, it should be noted that a robust scientific review process should facilitate efficiency in the much more expensive efforts conducted alongside or downstream from the SRC/IRB process. Overall, study activation, including IRB, contracting, budgeting, and ancillary reviews, can take between 90 and 120 days, or more, representing hundreds of hours of investment. This type of investment should be made for only studies with strong scientific merit. Even more importantly, at the end of the study, poorly designed aims, study design, and analytic methods will lead to ineffective efforts and failure to have results to report. If a study is abandoned late in study activation, opens but does not accrue participants, accrues but does not achieve target enrollment, or achieves underpowered enrollment targets, resources and the involvement of human participants will not have been effectively, appropriately, and arguably ethically employed. Institutional decisions about investments and commitments to SRC processes must take these issues into consideration.

### Limitations

This study had several limitations. Sites that volunteered to participate may not be representative of ethics review processes at academic medical research organizations. Six of the 10 participating sites had extant SRC processes that were largely aligned with the prioritized criteria of the recommended framework. This reduced the likelihood of detecting a difference between the study periods and may not reflect the larger group of organizations.

Only a subset of participating sites, SRCs, and protocols could be included in statistical analyses for the primary and secondary outcomes. Some SRCs did not implement a modification within the study period, others had no or very few eligible protocols during intervention, and others did not provide high-quality study data. Additionally, two high-volume sites that implemented a modification did so without the authority to require stipulations. A longer intervention period, more time for implementing modifications, and ensuring robust implementation could mitigate these limitations in future studies.

Meaningful sub-analyses by type of site (no extant SRC, single SRC, multiple SRCs) were beyond the scope and available data of this initial study. For feasibility analyses, substantial variation in SRC alignment within each type made it difficult to draw conclusions. For statistical analyses, neither of the two sites with multiple SRCs met criteria for inclusion in quality or efficiency analyses, leaving only two site types. The two high-volume sites that implemented a modification without authority to require stipulations were in the same site type; however, because only two of the four sites in the type did not require SRC stipulations to be addressed, there was no clear correlation between site type and SRC authority and any detectable difference would be uninterpretable or spurious. For qualitative analyses, which were conducted at the site level, to further subdivide 10 sites into 3 types would yield insufficient subsample sizes to draw conclusions by type.

Finally, this study focused on short-term outcomes. A longer tracking period could provide important insights about longer-term outcomes, such as whether clinical research protocols reviewed by SRCs are more likely to be completed, achieve accrual goals, or disseminate results. To facilitate future research in this area, all study-related tools created by Tufts CTSI are available upon request.

### Conclusions and Recommendations

The recommended SRC framework is feasible to implement across diverse organizations, provided that key barriers to full implementation are addressed. Although not conclusive, sites perceived positive impact on their protocol quality. During intervention, IRBs appeared to focus less on operational feasibility and to deliberate longer on protocols that may have needed more attention: SRC-reviewed protocols and those remaining under review for more than 3 weeks.

Lessons learned from this study include the importance of a strong institutional mandate, with substantial efforts to engage local leaders and stakeholders, or an external requirement, such as from NIH, to implement an SRC process. SRCs need clear authority to require investigators to address stipulations, or institutions will need IRBs to adopt an enforcement role. Across diverse AHCs, optimal benefit will require adapting the recommended processes to local contexts while maintaining the authority reflected in the spirit of the recommended framework.

## Supplementary material

For supplementary material accompanying this paper visit http://dx.doi.org/10.1017/cts.2019.439. click here to view supplementary material
