# Supplementary material for: Scientific Review Committees as part of institutional review of human participant research: Initial implementation at institutions with Clinical and Translational Science Awards
Source: J Clin Transl Sci. 2020 Jan 27;4(2):115–24. doi: 10.1017/cts.2019.439 (PMC7159811; doi:10.1017/cts.2019.439)
Supplement: Supplementary file 1 [file S2059866119004394sup001.docx]

**Supplemental Materials**

**Supplemental Figure 1. Alignment of Scientific Review Committee processes with Prioritized Criteria at Baseline and Intervention, by Type of Site**

0-30 points possible

**
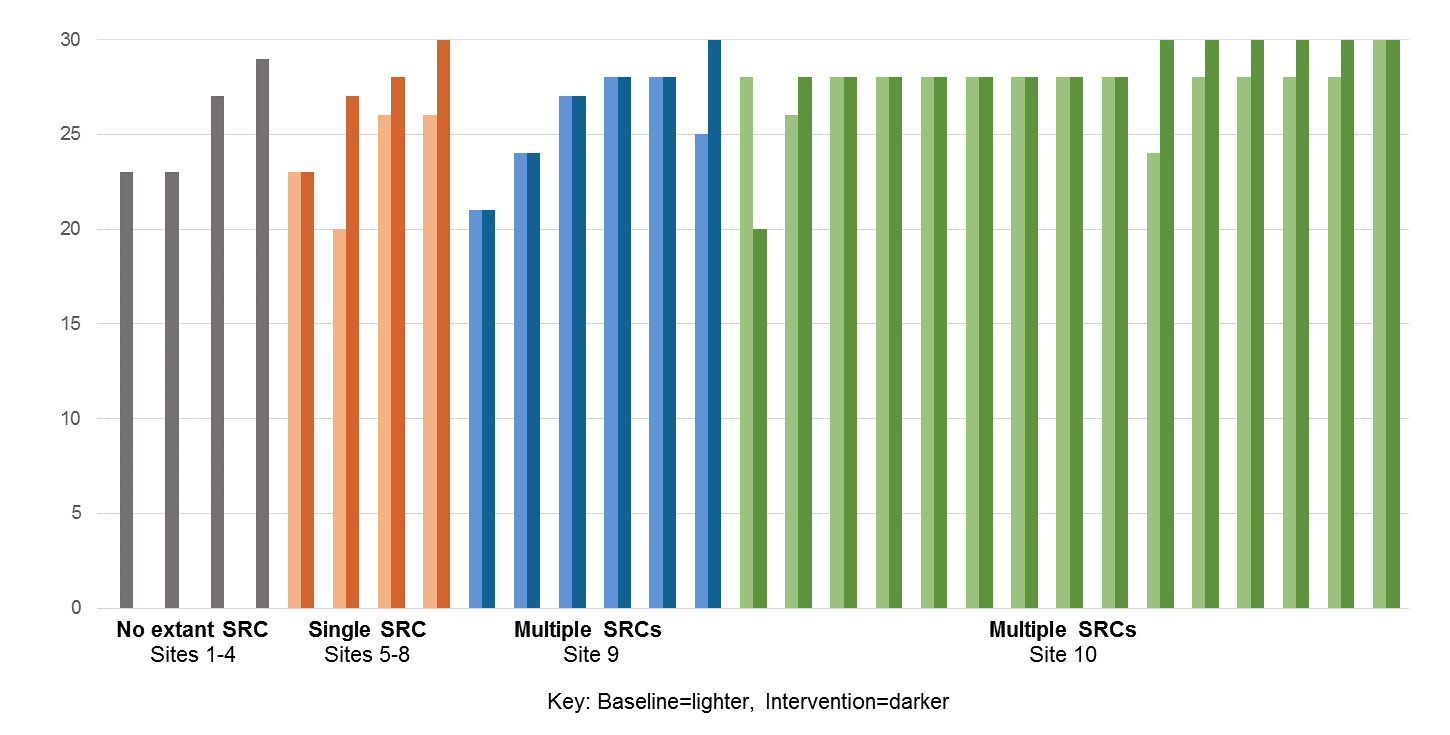
**

Intervention data are missing for one criteria at one SRC (2 points). Sites with no extant SRC at baseline do not have data for that period. At one site, two baseline SRCs merged into a single intervention SRC; the baseline score represented is the average of the two baseline SRC individual scores.

| **Supplemental Table S1. Recommended Scientific Review Committee Process Criteria, Study Priorities, and Alignment Scoring** | | |
| --- | --- | --- |
| **Recommended criterion** | **Prioritized by study?** | **Alignment score** |
| **Review content (6 points)** | |  |
| Review all protocols for scientific quality | 🗸 | 3 |
| *Excludes centrally reviewed protocols if central IRB has a SRC process* |  |  |
| Review all protocols for local operational feasibility | 🗸 | 3 |
| Determination that PI has necessary skills, experience, and time | - | n/a |
| **Protocol eligibility (6 points)** | |  |
| All protocols are eligible for SRC review or exemption criteria are limited to recommended types of protocols | 🗸 | 4 |
| Protocols that fit criteria for exemption can still be reviewed (includes SRCs that do not exempt protocols) | 🗸 | 1 |
| IRB and/or institutional officials may forward to the SRC any protocol at any time | 🗸 | 1 |
| **Reviewers per protocol (6 points)** | |  |
| Chair member | - | n/a |
| Coordinator member | - | n/a |
| Three or more medical/scientific reviewer members | - | n/a |
| - At least 1 medical reviewer | 🗸 | 2 |
| Statistician not also acting as a medical reviewer | 🗸 | 2 |
| Content experts as needed | 🗸 | 2 |
| **Reviewer qualifications (6 points)** | |  |
| Have requisite expertise | 🗸 | 2 |
| Not on research team | 🗸 | 1 |
| No conflict of interest | 🗸 | 1 |
| Is available to perform review in timely manner | 🗸 | 1 |
| Is willing to undertake the task | 🗸 | 1 |
| **Related institutional policies (6 points)** | |  |
| Coordination of the SRC with the IRB | 🗸 | 3 |
| IRB has access to SRC review | 🗸 | 3 |
| **Operational procedures (0 points)** |  |  |
| SRC-exempt protocols submitted directly to the IRB with no investigator action | - | n/a |
| SRC-eligible protocols may receive recommendations for major or minor revisions prior to SRC review | - | n/a |
| SRC-reviewed protocols may be returned to the investigator repeatedly prior to approval | - | n/a |
| SRC-approved protocols are submitted to the IRB directly with no investigator action | - | n/a |
| SRC meetings are conducted regularly provided there is a quorum and protocols to review | - | n/a |
| SRC quorum is (at minimum) three medical/scientific reviewers, one statistical reviewer, SRC Chair, and coordinator present | - | n/a |
| If a meeting cannot be convened at the regular time, an alternate time may be proposed and agreed upon within the same week | - | n/a |
| If the SRC Chair is not available for the meeting, the Chair would nominate a Deputy Chair | - | n/a |
| SRC recommendations are made by majority vote | - | n/a |
| Only members present for the majority of the meeting and for critical deliberations can vote | - | n/a |
| If a reviewer is unable to attend, but has reviewed the relevant information, consider his/her input deliberations | - | n/a |
| An attempt is made to achieve consensus in all decisions | - | n/a |
| **Informatics support (0 points)** | |  |
| Communication between SRC staff | - | n/a |
| Submission of protocols | - | n/a |
| Time stamping key points in the workflow | - | n/a |
| n/a=criterion that was not prioritized by the study | | |

| **Supplemental Table S2. Challenges for operationalizing a new process*** | |
| --- | --- |
| **Readjusting staffing** | |
|  | It's taking something that was done at [Unit A] and now it's housed at [Unit B], and so you have to figure out who's going to staff it and compensate them. *− Implementation Point Person* |
|  | And so [the biostatisticians] get some of their funding from the CTSA and some from [other sources] … There's a tension there with everything else that they're trying to do because they're not 100% under the CTSA. …The biostatistics review has been the problem for sure. *− SRC Chair* |
| **Training those impacted** | |
|  | I do think that we could have done a better job in our office with our board, kind of walking them through…the first [protocols with SRC review] that they came through…[to] see if there were any questions. − *IRB Chair* |
|  | [Research] staff, they pushed back as well, just in timeliness of reviews and it changed how…their old process worked, so making sure that everybody was aware of the process, making sure everybody was trained. – *Implementation point person* |
|  | And then the question is: Are they going to get confused between the grant [proposal] and the protocol? …So you really don't have anything from NIH that says ‘We've reviewed your protocol.’*– Local SRC champion* |
| **Aligning expectations** | |
|  | I think what we've done is…to try to set expectations for exactly that problem where things would come back [from the SRC] and then start the [IRB] pre-review after they've already been through an extensive review, which might have been quiet exhausting for the PI the first time around… *– IRB Chair* |
|  | There’s some debate. ...Many of our [SRC] reviewers had experience of the IRB, and we have to remind ourselves that this is not the IRB, so this is not about subject protection. Many others of us have served on grant reviews [for funding decisions], and we have to remind ourselves this is not a grant review session either. *– Local* *SRC champion* |
| **Implementing new information technology systems or workflows** | |
|  | [O]ur IRB system is not the most user friendly in general, and then I had to figure out how to jet stream my [SRC] trials because I could see then 30 million trials. …And we did have to, of course, change the IRB [electronic] system to permit this added layer of review, and that was a very complicated IT situation. And I will tell you, it's not perfect. *– IRB Chair* |
| *Among seven sites that addressed operational challenges. The challenge(s) experienced at each site differed due to local context and the extent of the modification undertaken. Each challenge was reported by at least two sites. | |

| **Supplemental Table S3. Anticipated barriers that did not materialize as expected** | |
| --- | --- |
| **Resistance from investigators**  *Anticipated at ten of ten sites* | |
|  | I was very concerned that people were going to not be happy when they’re told something [about a protocol] is not right. But what we have found is… it’s usually tends to be younger, newer people to [Institution] and they just don’t know how to navigate and get the resources they need. And so, actually, they’ve been grateful for the help. *– Local SRC champion* |
|  | So far, I don’t think many people have been affected by it, …so if we then did…make it to include all human subjects [research] then we might face more resistance. I just don’t know. *– Local SRC champion* |
|  | …Our institution did implement an extremely facile mechanism for achieving the SRC review with ad hoc, as needed spontaneous meetings such that investigators do not have to wait for weeks or a month to get their SRC review done. I think that that is probably why we have not had difficulties with investigator complaints et cetera. *– IRB Chair* |
| **Adding delays to review time** | |
|  | I mean there’s a certain number of investigators that…don’t understand why we’re doing scientific review. …They have the perception that it could create a delay. They feel that they’re pretty well established investigators and, you know, they don’t recognize…the added value of SRC. *– Local SRC champion* |

| **Supplemental Table S4. External reviewer assessments of protocol quality, by period** | | | |
| --- | --- | --- | --- |
| **Reviewer type** | **Overall assessment score* (mean, SD)** | | **p-**  **value** |
|  | Baseline | Intervention |  |
| All eligible protocols | N=59** | N=58** |  |
| Content reviewer | 4.4 (2.0) | 4.8 (1.5) | 0.2 |
| Statistical reviewer | 4.6 (1.7) | 4.5 (1.7) | 0.8 |
| Combined reviewers | 4.5 (1.7) | 4.6 (1.4) | 0.6 |
| Protocols with requirement to address SRC stipulations | N=17 | N=17 |  |
| Content reviewer | 4.5 (2.1)) | 4.9 (1.2) | - |
| Statistical reviewer | 5.1 (1.6) | 4.9 (1.6) | - |
| Combined reviewers | 4.8 (1.7) | 4.9 (1.2) | - |
| SD=Standard Deviation  *Possible scores: 1-9.  ** Two protocols in the original sample were outside the eligibility time window and a third was removed to maintain balanced representation of sites at baseline and intervention. | | | |
